# Combining a Fuzzy Matter-Element Model with a Geographic Information System in Eco-Environmental Sensitivity and Distribution of Land Use Planning

**DOI:** 10.3390/ijerph8041206

**Published:** 2011-04-18

**Authors:** Jing Zhang, Ke Wang, Xinming Chen, Wenjuan Zhu

**Affiliations:** 1 Institute of Remote Sensing & Information System Application, Zhejiang University, Hangzhou, 310029, China; E-Mail: zj1016@163.com; 2 Land Consolidation Center in Zhejiang Province, Hangzhou, 310007, China; E-Mail: chen-000@163.com; 3 School of Geography, University of Leeds, Leeds, LS2 9JT, UK; E-Mail: w.zhu09@leeds.ac.uk

**Keywords:** fuzzy matter-element model, geographical information system, evaluation of eco-environmental sensitivity, Yicheng City

## Abstract

Sustainable ecological and environmental development is the basis of regional development. The sensitivity classification of the ecological environment is the premise of its spatial distribution for land use planning. In this paper, a fuzzy matter-element model and factor-overlay method were employed to analyze the ecological sensitivity in Yicheng City. Four ecological indicators, including soil condition,, water condition,, atmospheric conditions and biodiversity were used to classify the ecological sensitivity. The results were categorized into five ranks: insensitive, slightly sensitive, moderately sensitive, highly sensitive and extremely sensitive zones. The spatial distribution map of environmental sensitivity for land use planning was obtained using GIS (Geographical Information System) techniques. The results illustrated that the extremely sensitive and highly sensitive areas accounted for 14.40% and 30.12% of the total area, respectively, while the moderately sensitive and slightly sensitive areas are 25.99% and 29.49%, respectively. The results provide the theoretical foundation for land use planning by categorizing all kinds of land types in Yicheng City.

## 1. Introduction

Eco-environmental sensitivity refers to the degree of sensitivity of an ecosystem to human activity, which reflects the potential for antropogenic ecological imbalances and eco-environmental problems [1]. Eco-environmental sensitivity of land use planning is a comprehensive index which serves as a basis for land use planning and eco-environmental management. It includes eco-environmental quality, population load, reasonable degree of land use and the level of economic development. The eco-environment directly affects the land use pattern and the opposite is true where unreasonable land use planning is responsible for the deterioration of the eco-environment.

At present, research on eco-environmental sensitivity has developed rapidly and has been put into practice. Cameron *et al.* carried out classification and evaluation research for animal-environment sensitivity [2]. Satti *et al.* investigated agriculture-water management in swamps by assessing the ecological sensitivity of climate, soil and crops [3]. An eco-environmental sensitivity method was applied to Gansu Province (China) and eco-environmental sensitivity was defined as the possibility of ocurrence of eco-environmental problems in the region, influenced by natural factors. A high sensitivity region was found where eco-environmental problems might occur easily due to human activities [4]. Using GIS, some scholars have conducted evaluations on the sensitivity of water and soil erosion, land degeneration, desertification and then classified the regional land use types; these results provided the scientific basis for ecological construction and sustainable development in Jilin Province (China) [5]. Li *et al.* evaluated the sensitivity of soil erosion, stony desertification and the eco-environment and divided the results into five grades: slighter, slight, middle, bad and worse [6]. In line with the existing issues of the land ecological environment in Shanxi Province (China) and the theory of eco-environmental sensitivity, some scholars selected soil erosion, geological disasters, biodiversity and the ecological environment as ecologically sensitive factors, and carried out a superimposition analysis using the ArcGIS software to create special charts of five single essential factors according to the first rank of ecologic sensitivity for an ecologically sensitive area in this province [7]. However, to date sensitivity analyses have only emphasized certain fields such as drainage basins and natural disaster factors at a large scale [8,9]. Correlative studies of eco-environmental sensitivity and land planning are rarely carried out for small scale regions [10,11]. Besides, the published studies mostly adopted qualitative methods and used a GIS module as a tool to display the results, but these studies did not integrate quantitative methods and GIS with the eco-environmental sensitivity.

GIS based integrated quantitative methods provide an excellent framework for data capture, storage, synthesis, measurement, and analysis, all of which are essential for analyzing eco-environmental sensitivity. To provide an objective result for eco-environmental sensitivity evaluation, in this study a new environmental numerical evaluation model was developed and applied using a fuzzy matter-element (FME) model and GIS. With the support of the FME model and GIS, the study examines the overall land use planning of Yicheng City (2006–2020) as an integrated system. The objectives of this study were: (1) to develop an environmental numerical evaluation model supported by FME, (2) to establish a synthetic eco-environmental sensitivity index, (3) to establish the distribution of eco-environment sensitivity via GIS.

## 2. Study Area

**Figure 1.**
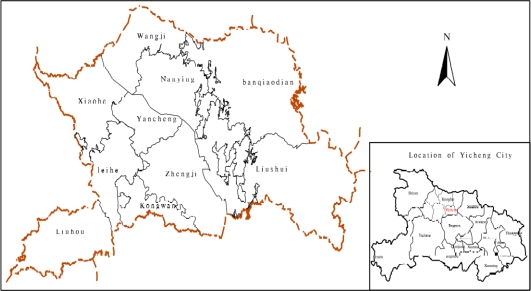
Location of the study area.

The study site is Yicheng City (Figure 1), which is located at 111°57’–112°45’ E, 31°27’–31°54’ N in the north of Hubei Province. It is located to the east of Zaoyang and Suizhou, south of Zhongxiang and Jingmen, west of Nanzhang and north of Xiangyang. The distance from the east to the west is 76 kilometers, the width is 53 kilometers and the total area is 2,045 square kilometers (Figure 1). It is characterized by a humid subtropical climate, with abundant heat and light. Annual average temperature is 16 °C, annual rainfall is 850–1,000 mm, annual sunlight is 1,932 hours and annual average relative humidity is 76% [12].

## 3. Methodology

### 3.1. Establishment of the Evaluation Index System

Based on the idea of sustainable development and the question driven model of the Organization for Economic Co-operation and Development (OECD), this paper puts forward a synthetic evaluation index of “driving forces—pressure—appearance—influences—respond to”.

Choosing a proper evaluation index system was the basis of our eco-environmental analysis. Through analysis of the current conditions of the regional eco-environment, several ecological elements closely related to the ecological sensitivity were determined and employed. These were soil erosion, water conditions, atmospheric conditions and biodiversity. Via multi-factor synthetic appraisement and clustering methods integrated with Data Processing System (DPS) and GIS techniques, the paper established the distribution and division of eco-environmental sensitivity for land use planning.

### 3.2. The Evaluation Index and Gradation

There are many physical and human factors that affect eco-environmental sensitivity. Choosing the appropriate indexes plays a vital role in environmental sensitivity assessment. Based on the eco-environmental characteristics of the region, an evaluation index system with four major groups A-D was established for Yicheng City. When carrying out calculation of indexes, the order is from the bottom index layer (D layer) to highest layer (A layer) of eco-environmental sensitivity. The layers include four categories (soil conditions, water conditions, atmospheric conditions and biodiversity), 19 subcategories and a total of 40 factors. The evaluation indexes and grades of eco-environmental sensitivity for land use planning are presented in Table 1.

### 3.3. Evaluation Methods and Model of Synthetic Eco-Environmental Sensitivity

#### 3.3.1. Fuzzy Matter-Element Model

##### (1) Compound fuzzy matter-element model

Fuzzy matter-element analysis was adopted to evaluate the environmental quality of land use planning. If the name of the design object is regarded as a matter-element character and the value of the objective function is regarded as a mathematical measurement value, the matter-element form of the multi-objective will become in turn a three-factor group. If the design variable x has fuzzy character, the variable is named fuzzy matter-element. If the evaluated example M has n characters C_1_, C_2_,…, C_n_ and the corresponding variable x_1_, x_2_, x_3_,…, x_n_, the model R is named a fuzzy matter-element of dimension n. M objects and fuzzy matter-elements of n dimensions make up the matter-element form of the multi-objective R_mn_ as follows [13]:

$$R_{mn}=\left[\begin{array}{lllll} & M_{1} & M_{2} & \cdots & M_{m}\\ C_{1} & x_{11} & x_{21} & \cdots & x_{m1}\\ C_{2} & x_{12} & x_{22} & \cdots & x_{m2}\\ \cdots & \cdots & \cdots & \cdots & \cdots \\ C_{n} & x_{1n} & x_{2n} & \cdots & x_{mn} \end{array}\right]$$

where R_mn_ is the matter-element form of the multi-objective, M_i_ is the its object (i = 1, 2,……, m); c is its character (j = 1, 2,……, n); x_ij_ is the corresponding design value.

##### (2) The principle on the excellent dependent degree

If x is an ordinary mathematical function expression, according to the optimization principle, an excellent dependent degree function is as follows [13]:

(1) $$\text{The}\;\text{ideal}\;\text{maximum}\;u_{\text{ij}}=x_{\text{ij}}/\text{max}\;x_{\text{ij}}$$

(2) $$\text{The}\;\text{ideal}\;\text{maximum}\;u_{\text{ij}}=\text{min}\;x_{\text{ij}}/x_{\text{ij}}$$

where x_ij_ is the excellent dependent degree, min x_ij_ is the ideal minimum of the evaluated index while max x_ij_ is the ideal maximum of the evaluated index.

Therefore, a fuzzy matter-element of excellent dependent degree $\bar{R_{mn}}$ is as follows [13]:

$$R_{mn}=\left[\begin{array}{lllll} & M_{1} & M_{2} & \cdots & M_{m}\\ C_{1} & u_{11} & u_{21} & \cdots & u_{m1}\\ C_{2} & u_{12} & u_{22} & \cdots & u_{m2}\\ \cdots & \cdots & \cdots & \cdots & \cdots \\ C_{n} & u_{1n} & u_{2n} & \cdots & u_{mn} \end{array}\right]$$

##### (3) Standard fuzzy matter-element and fuzzy matter element of different squares

The standard fuzzy matter-element is confirmed by the minimum and the maximum on the relative membership grade of every evaluated index. If the squares of every item are different from the standard fuzzy matter-element R_0n_ and fuzzy matter element R_mn_ should be Δ*_ij_* = (*u_oj_* – *u_ij_*)^2^, (*i* = 1,2,⋯, *n; j* = 1,2,⋯, *m*), the fuzzy matter-element model can be written as:

$$R_{mn}=\left[\begin{array}{lllll} & M_{1} & M_{2} & \cdots & M_{m}\\ C_{1} & \Delta_{11} & \Delta_{21} & \cdots & \Delta_{m1}\\ C_{2} & \Delta_{12} & \Delta_{22} & \cdots & \Delta_{m2}\\ \cdots & \cdots & \cdots & \cdots & \cdots \\ C_{n} & \Delta_{1n} & \Delta_{2n} & \cdots & \Delta_{mn} \end{array}\right]$$

##### (4) Weight of evaluation factors

This paper introduces the entropy method to confirm the weight of evaluation factors. The entropy method is a systematic analysis evaluation method to treat complex and multi-index systems quantitatively, which can overcome the irrationality of evaluation results resulting from subjective factors. The method can reduce subjective distributions and make the results more practical [13]. The detailed analytical process is as follows [14]:

- Assuming the amount of the evaluated objects is m, the amount of the evaluated index is n, to establish the estimation matrix R:
  (3) $$R={\left(r_{\text{ij}}\right)}_{\text{mn}},(i=1,2,\cdots ,n;j=1,2,\cdots ,m)$$
- The corresponding estimation matrix R is ranked and obtained the total ranking matrix B. The factor of matrix as:
  (4) $$b_{\text{ij}}=r_{\text{ij}}-r_{\text{min}}/r_{\text{max}}-r_{\text{min}}$$
  where r_max_ and r_min_ respectively represent the best and the worst of different things in the same evaluation index, which means the bigger, the more satisfied or the smaller, the more satisfied.
- According to the concept of entropy, it defines the entropy including m evaluation indexes and n evaluation indexes:
  (5) $$H_{I}=-\sum_{j=1}^{m}f_{\text{ij}}\;\text{ln}\;f_{\text{ij}}/\text{ln}\;m,(i=1,2,\cdots ,m;j=1,2,\cdots m)$$
  The concept of f_ij_ is as follows:
  (6) $$f_{\text{ij}}=b_{\text{ij}}/\sum_{j=1}^{n}b_{\text{ij}}$$
  When f_ij_ = 0, Lnf_ij_ is inanition, so the calculation must be corrected. The concept is as follows:
  (7) $$f_{\text{ij}}=1+b_{\text{ij}}/\sum_{j-1}^{m}\left(1+b_{\text{ij}}\right)$$
- Calculate weigh of index w and entropy weight w_i_:
  (8) $$W={\left(w_{i}\right)}_{1\times m};w_{i}=\left(1-H_{i}\right)/(n-\sum_{i-1}^{n}H_{i})$$
  and $\sum_{i=1}^{n}w_{i}=1$

##### (5) Euclid approach degree and synthetic evaluation

In fuzzy theory, the Euclid approach degree is commonly used to describe the degree of proximity of two scenarios or two samples. According to the degree of dependence, the scheme can be judged as to whether it is good or not. The larger the degree of dependence is, the better the scheme. Therefore, this paper applied the Euclid approach degree of the evaluated fuzzy matter-element to determine the compositor of excellent and inferior and to attain the grade classification of eco-environmental quality of the evaluated samples. The advantage of Euclidean proximity is that not only is it convenient, but it also overcomes the disadvantage of the former weight evaluation model [14]. The equation for calculating the degree of dependence PH_j_ is as follows:

(9) $${\text{PH}}_{j}=1-\sqrt{\sum_{i=1}^{m}w_{i}\Delta_{\text{ij}}},j=1,2,\cdots ,m$$

where PH_j_ is the dependent coefficient.

Let R_PH_ be the compound matter-element of the scheme fuzzy matter-element sub-object, then:

$$R_{PH}=\left[\begin{array}{lllll} & M_{2} & M_{2} & \cdots & M_{m}\\ {\text{PH}}_{j} & {\text{PH}}_{1} & {\text{PH}}_{2} & \cdots & {\text{PH}}_{m} \end{array}\right]$$

#### 3.3.2. The GIS Technique Route and Factor-Overlay Method

**Figure 2.**
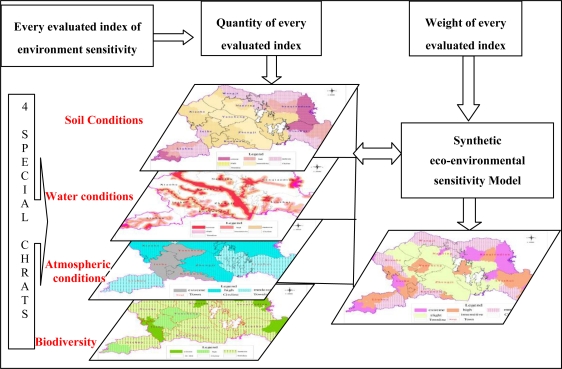
Technique of synthetic eco-environmental sensitivity for land use planning.

In order to quickly obtain the calculated results from analysis function in ArcGIS 9.3, descriptive level of information needs to be converted into quantitative indexes and build index system (Table 1). The calculation process of the paper was based on vector data. Using ArcGIS 9.3 and the fuzzy matter-element model, every evaluated unit of soil, water, atmosphere and biodiversity were given a corresponding grade index and converted into the basic evaluation unit to form the regional eco-environmental information system database, and then four special sensitive charts were obtained. These were the soil conditions, water conditions, atmospheric conditions and biodiversity charts types, which were reprojected onto the standard projection system. Taking advantage of the space overlay function in advanced GIS technology and selecting the administration village as the basic unit, this paper carried out the superimposition analysis to produce special charts of four single essential factors according to the first rank of ecological sensitivity, to calculate the comprehensive index of eco-environmental sensitivity in every space unit and to confirm scientific and reliable evaluation grades. Then the results were carried through spatial clustering to merge and regulate the basic spatial units and obtained the ecological environment sensitivity of the research area [15,16]. The technique of synthetic eco-environmental sensitivity for land use planning using GIS and the factor-overlay method is shown in Figure 2.

#### 3.3.3. Operation Model of Multi-Factor Vector Data

1. Due to its ability to assign proper weights to various factors in complex systems, the eco-environment system was suitable for use in the entropy method, which was introduced in Section 3.3.1.
2. The operation model of multi-factor vector data was constructed. Synthetic evaluation of ecological sensitivity on land use planning should synthetically consider every kind of evaluation index, including soil conditions, water conditions, atmospheric conditions and biodiversity. The formulation below was adopted to calculate the index grade of ecological sensitivity and to comprehensively evaluate the whole environmental sensitivity on the basis of the index grades. The formulation was as follows [15]:
  (10) $$G_{i}=\sum_{i=1}^{n}W_{j}\times g_{i}$$
  where G_i_ was the synthetic index value of every factor on B layer, g_i_ was the value of each index, w_j_ was the weight of each index, and n was the total number of indices, i = 1, 2, 3,…, n.

By means of GIS techniques and the factor-overlay method, we obtained the synthetic distribution map of environmental sensitivity for land use planning, which was helpful to lay out all kinds of land types in general land use planning to produce a smaller influence or non-negative influence. It presented a sufficient and authoritative map for the organizers of general land use planning, and supplied the scientific basis for the space distribution on every kind of land type.

## 4. Results and Discussion

### 4.1. Sensitivity of Soil Conditions

**Figure 3.**
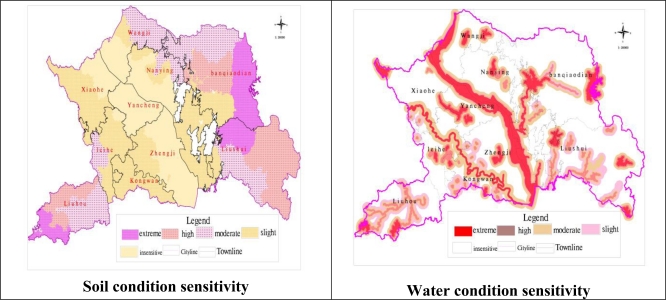

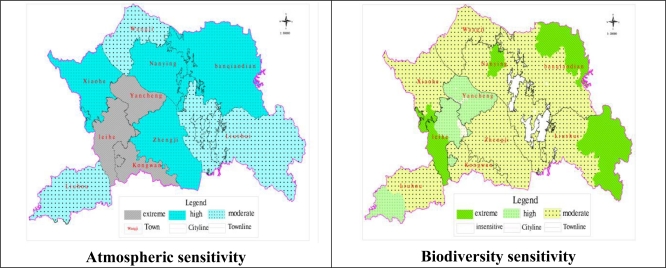
Sensitivity evaluation grade maps.

Soil conditions are controlled by factors including soil and water loss, soil quality, land use/land cover, influence of the intensity of land use, *etc*. Soil quality is crucial for soil conditions and it varies with landforms and the environment. From the spatial distribution of soil-erosion sensitivity (Figure 3), it was deduced that the extremely sensitive area covered 20,452.99 hm^2^ and accounted for 9.97% of the total area in Yicheng City, corresponding mainly in the high mountainous region where the landform was very complicated. The highly sensitive area was 28,535.72 hm^2^ and accounted for 13.91%, which was distributed in low mountains and high hills region. The moderately sensitive area was 38,218.58 hm^2^ and accounted for 18.63%, which was distributed in low valley regions. The slightly sensitive area was 61,215.37 hm^2^, accounted for 13.91%, and was distributed in low valleys and flat regions. The insensitive area was 56,722.69 hm^2^, it accounted for 27.65%, which was mainly distributed in the flat region where hypsography was very mild (Figure 3 and Table 2).

### 4.2. Sensitivity of Water Conditions

Water conditions were determined by various factors such as water quality, distance to water sources, quality of protection layer, and water exchange intensity. Water quality and distance to water sources were the key factors. The extremely sensitive water condition area mainly involved the towns of Yancheng, Wangji and Nanying which were the nearest to the Hanjiang River, Man River, lakes, reservoirs, wetland areas and so on. The water resources of these regions were extremely rich and were the main origin of the centralized subsistence water. The highly sensitive area was located between the common sensitive section of the river and the highly sensitive section of the river, which was rich in high quality water resources used for human consumption, industry and agriculture; the corresponding river system included the buffer region up to 500 meters away from the water source. The moderately sensitive area was the buffer region one kilometer away from the water source, involving in part the the Leihe development region and the town of Kongwan. The insensitive region referred to the region without a big lake or reservoir area, mainly distributed in the region of the towns of Liuhou and Liushui, where water resources were relatively scarce and water quality was relatively inferior.

### 4.3. Sensitivity of Atmospheric Conditions

Atmospheric conditions were affected by several indicators, including air quality, content of SO_2_ (daily mean), content of NO_2_ (daily mean), pollution burden and so on. Air quality had a major effect. The evaluation results for the surrounding environment (Table 3) indicated that the extremely sensitive area was 31,265.67 hm^2^ and accounted for 15.24% of the total area in Yicheng City, and laid mainly in Yancheng Town, the Leihe development zone and Kongwan Town. This region is the central construction and industry zone. The highly sensitive area was 97,184.32 hm^2^, accounted for 47.37%, and included Xiaohe, Nanying, Banqiaodian and Zhengji towns, which are distributed on the periphery of the extremely sensitive area. The moderately sensitive area was 76,695.35 hm^2^ and accounted for 37.39%. It include Liuhou Town, Wangji Town and Liushui Town, which constitute the main food supplying region and are separated from the extremely sensitive area.

### 4.4. Biodiversity Sensitivity

Biodiversity was influenced by factors such as abundance of biological resources, degree of social and economic development and so on. The evaluation of results for the biodiversity environment (Figure 3) showed that the extremely sensitive area was 43,445.33 hm^2^ and accounted for 21.18% of the total area in Yicheng City, mainly including key points of the landscape, ecological protection areas, conservation areas, protective forests along the river and the water maintenance forest. The highly sensitive area was 17,770.25 hm^2^ and accounted for 8.66%, which was distributed in Xiaohe Town, Liushui Town, Liuhou Town, Banqiaodian Town and Zhengji Town. The region should be regarded as a lasting and effectively protected area according to the demands of society, economy and sustainable development. It was abundant in arable land and prime farmland, and the scale was central and the quality was superior. The moderately sensitive area was 143,929.76 hm^2^ and accounted for 70.18%, mainly included important urban-rural construction land and industrial land.

### 4.5. Synthetic Evaluation and Space Distribution of Eco-Environmental Sensitivity

**Figure 4.**
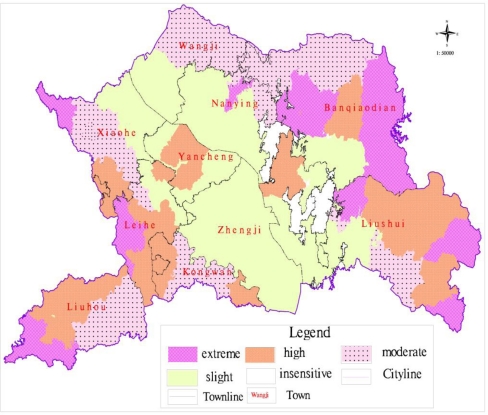
Grade map of synthetic eco-environment sensitivity in Yicheng City.

According to the practical situation in Yicheng City, based on the results of a series of single evaluated indexes, the weights of all elements in a level of the hierarchy relative to a whole level directly above could be obtained, was totally ranked, and was carried from the upper layer to the lower. After the above analytical process, the weight of each evaluation factor was determined for an integrated evaluation of the eco-environmental sensitivity of Yicheng City (Table 5). In order to calculate the synthetical index of ecological sensitivity, the factor-overlay method with GIS technique was adopted to confirm scientific and reliable grades, ultimately the distribution map of synthetic eco-environmental sensitivity on land use planning was obtained, which could comprehensively reflect the ecological environment for Yicheng City (Figure 4 and Table 6).

The spatial distribution of synthetic sensitivity showed that the extremely sensitive area was 29,540.93 hm^2^ and accounted for 14.4% of the total area in Yicheng City, and that it laid mainly in the eastern low mountain area and the southwest region, including parts of Liushui Town, Banqiao Town, Liuhou Town and Leihe Town. The ecological sensitivity of the region was extremely high, and the external factor disturbance not only had a quite intense influence on the region, but also affected the peripheral region and subsequently resulted in the destruction of the entire ecological environment system. Therefore, this area should be considered as the key protected region.

The highly sensitive area was 61,789.78 hm^2^, accounted for 30.12% of the total and was located around the Han River and Man River. The areas covered the major part of Xiaohe Town, Wangji Town, Yancheng Town, and Nanying Town, where there was high soil and biodiversity sensitivity. The ecological sensitivity of the region was relatively high, and it would be important to maintain the extremely sensitive area function and protect the total ecological environment of Yicheng City. As a result, close attention should be paid to the highly sensitive area in land use planning.

The moderately sensitive area was 38218.58 hm^2^ and accounted for 25.99%. It mainly included the towns of Wangji, Banqiaodian, Kongwan, Xiaohe and Liuhou, which were affected by atmospheric and biodiversity sensitivity. Environmental conditions in the area was relatively good and it was rational to carry on appropriate land exploitation, which could help to develop the forestry and fruit industry aiming at building up the basic forestry and fruit industry of Yicheng City.

The slightly sensitive area was 60,497.36 hm^2^, it accounted for 29.49% and included parts of the region of Nanying Town, Yancheng Town and Zhengji Town, which were influenced by water and atmospheric sensitivity. The area was the crucial land use region, and it was also a major base of grain planting in Yicheng City. Hence, in the process of land development and utilization, it is necessary to improve land productivity and usage efficiency. The slightly sensitive area was suitable for high-intensity exploitation and construction of large enterprises which exert a slight influence on the ecological environment, but the exploitation style and the possible environmental pollution should be considered.

## 5. Conclusions

This article applies a fuzzy matter-element model and a GIS vector overlay technique to carry out an ecological sensitivity evaluation and distribution for a small region. It overcomes some of the disadvantages of conventional techniques, such as the long period, complex manipulation, and fragmented landscape elements [17]. The results showed that:

1. It was proven that the spatial-overlay analysis for ecological sensitivity research was efficient and accurate. GIS was able to quickly and accurately extract a variety of basic information of ecological and environmental aspects, generate and update thematic maps of different ecological factors in different phases. Dynamic changes of the ecological environment obtained from the study area were very important to improve the eco-environmental management efficiency of the Land-Use Planning Department and Environmental Protection Department [18].
2. On the basis of the fuzzy set theory and the concept of Euclid approach degree, a fuzzy matter-element model for environmental impact assessment in general land use planning was established. A case study was carried out which the fuzzy matter-element model was used to assess the eco-environmental quality in a small scale region. The study results showed that the fuzzy matter-element method was practical and reliable. It applied a new method to environment impact assessment for general land use planning.

## Figures and Tables

**Table 1.** The evaluation index and grade system of eco-environmental sensitivity on land use planning.

**A layer**	**B layer**	**C layer**	**D layer (unit)**	**Insensitive**	**Slightly sensitive**	**Moderately sensitive**	**High sensitive**	**Extremely sensitive**
Eco-environmental sensitivity index	Soil conditions	Soil and water loss	Rainfall erosion (MJ·mm/hm^2^·h)	≤25	25∼100	100∼400	400∼600	≥600
			Vegetation type	Water, swamp, paddy-field	Forest, meadow, pour-cluster	Sparse shrub plain	Hungriness	Non-vegetation
			Grade and Slope (°)	0∼5	6∼10	11∼15	15∼25	≥25
			Soil texture class	Silty soil, silt	Sandy loam, silty clay, loam clay	Sandy soil, loam soil	Sandy soil, clay	Stone soil, sandy soil
		Soil quality	Soil type	Paddy soil	Damp soil	Yellow-Brown Soil	Purple soil	Calcareous soil
			Landform and physiognomy	Plain	Plain, hill	Hill	Hill, mountains	Mountains
			pH	≤5.5	5.5∼6.5	6.5∼7.5	7.5∼8.5	≥8.5
			Hg (ppm)	≤0.15	0.15∼0.30	0.30∼0.50	0.50∼1.0	1.0∼1.5
			Zn (ppm)	≤100	100∼200	200∼250	250∼300	300∼500
			Ni (ppm)	≤40	40∼50	50∼60	60∼70	70∼200
			HCHs ^(1)^ (ppm)	≤0.05	0.05∼0.15	0.15∼0.3	0.3∼0.5	0.5∼1.0
			Groundwater Depth(m)	≥200	100∼200	60∼100	40∼60	≤40
		Land use /land cover		Water, beach, swamp, paddy-field	Woodland, shrubberyland	Scanty-woodland, garden, meadow	Dry land, rural residential land	Non-vegetation land
		Influence intensity of land use		≤50	50∼60	60∼70	70∼80	≥80
	Water conditions	Water quality	Water sort	I	II	III	IV	V
			BOD ^(2)^ (mg/L)	2	3	5	10	80
			COD_Mn_^(2)^ (mg/L)	2	4	6	10	80
			Ammonia and nitrogen(mg/L)	0.1	0.2	1.0	2.0	8.0
			Cr∼(6+)(mg/L)	0.01	0.03	0.05	0.08	0.1
		Supply function of resource		Other used-water zone	Used-water zone of ecological forest and grass	Industrial used-water zone	Used-water zone of forest, fruit, animals, fishery	Drinking water zone, Water source zone
Index of eco-environmental sensitivity	Water condition	Exchange intensity of water		I	II	III	IV	V
		Distance to water source (m)		≤200	200∼400	400∼600	600∼800	≥800
		Quality of protection layer		I	II	III	IV	V
	Atmosphere condition	Content of SO_2_(Daily Mean)^(3)^ (mg/L)		≤0.05	0.05∼0.15	0.15∼0.20	0.20∼0.25	≥0.25
		Content of NO_2_(Daily Mean)^(3)^ (mg/L)		≤0.08	0.08∼0.09	0.09∼0.10	0.10∼0.12	≥0.12
		TSP ^(3)^ (mg/L)		≤0.12	0.12∼0.2	0.20∼0.30	0.30∼0.50	≥0. 50
		PM_10_^(3)^ (mg/L)		≤0.05	0.05∼0.10	0.11∼0.15	0.16∼0.25	≥0. 25
		Disposal ratio of industrial gas (%)		≥90	80∼90	70∼80	60∼70	≥60
		Pollution burden		Low	Relatively low	Middle	Relatively high	Quite high
		Population intensity(person/km^2^)		≤2,000	[2,000, 4,000]	[4,000, 6,000]	[6,000, 10,000]	≥10,000
	Biodiversity	High degree of non-living environment	Temperature of annual average(°C)	16	15	14	13	12
			Quantity of rainfall(mm)	1,000–1,050	1,050–1,100	950–1,000	900–950	850–900
		Abundance of biological resource	Protection Wildlife of national and province rank /marsh species	Unprotected species	Other protected species at province and regional level	Other protected species of nationality level	Marsh (without first and second level)	First and second level of nationality
			Cover ratio of vegetation (%)	25–30	20–25	15–20	10–15	5–10
			Biological diversity	≥1.8	1.6∼1.8	1.4∼1.6	1.2∼1.4	1.0∼1.2
		Degree of social and economic development	Average fixed assets investment	1	0.8	0.6	0.4	0.2
			Average net income (dollar)	1,500	1,200	1,000	850	780
			Engle coefficient; (%)	30	40	50	60	80
			Urbanization level; (%)	20–30	20–25	15–20	10–15	<10
Evaluation				1∼3	3∼5	5∼7	7∼9	9∼10

Note:

(1) Heavy metals are counted by element, which is applicable for cation exchange capacities greater than 5 cmol (+)/kg, and the unit of element content is ppm (10^−6^). If the content is less than or equal to 5 cmol (+)/kg, its standard value is half of value in the table; HCHs is hexachlorocyclohexane.

(2) BOD is Biochemical Oxygen Demand and COD_Mn_ is chemical oxygen demand. The units of BOD, COD_Mn_, ammonium and Cr∼(6+) are mg/L.

(3) TSP is Total Suspended Particular and PM_10_ is Particular matter less than 10 μm. The units of SO_2_, NO_2_, TSP, PM_10_ are mg/L (standard status) and time is to adopt daily average.

**Table 2.** Soil-erosion sensitivity evaluation results for Yicheng City.

**Type of sensitivity**	**Sensitivity index**	**Area (hm^2^)**	**Proportion (%)**
Insensitive	1	56,722.69	27.65
Slightly	3	61,215.37	29.84
Moderately	5	38,218.58	18.63
Highly	7	28,535.72	13.91
Extremely	9	20,452.99	9.97

**Table 3.** Atmosphere sensitivity evaluation results for Yicheng City.

**Type of sensitivity**	**Sensitivity index**	**Area (hm^2^)**	**Proportion (%)**
Moderately	5	76,695.35	37.39%
Highly	7	97,184.32	47.37%
Extremely	9	31,265.67	15.24%

**Table 4.** Biodiversity sensitivity evaluation results for Yicheng City.

**Type of sensitivity**	**Sensitivity index**	**Area (hm^2^)**	**Proportion (%)**
Moderately	5	143,929.76	70.16%
Highly	7	17,770.25	8.66%
Extremely	9	43,445.33	21.18%

**Table 5.** Weight of each eco-environmental evaluation index.

**A layer**	**Weight (w)**	**B layer**	**Weight (w)**	**C layer**	**Weight (w)**	**D layer**
Index of eco-environmental sensitivity	0.26	Soil conditions	0.2	Soil and water loss	0.2	Rainfall erosion (MJ·mm/hm^2^·h)
					0.2	Vegetation type
					0.25	Grade and Slope (°)
					0.35	Soil texture class
			0.32	Soil quality	0.24	Soil type
					0.18	Landform and physiognomy
					0.18	pH
					0.09	Hg (ppm)
					0.09	Zn (ppm)
					0.08	Ni (ppm)
					0.08	HCHs (ppm)
					0.08	Groundwater Depth (m)
			0.28	Land use /land cover		
			0.2	Influence intensity of land use		
	**0.24**	Water conditions	0.25	Water quality	0.55	Water sort
					0.12	BOD (mg/L)
					0.11	COD_Mn_ (mg/L)
					0.11	Ammonia and nitrogen (mg/L)
					0.11	Cr∼(6^+^) (mg/L)
			0.12	Supply function of resource		
			0.18	Exchange intensity of water		
			0.23	Distance to water source (m)		
			0.22	Quality of protection layer		
			0.28	Quality grade of air		
	**0.23**	Atmospheric conditions	0.14	Content of SO_2_(Daily Mean) (mg/L)		
			0.13	Content of NO_2_(Daily Mean) (mg/L)		
			0.12	TSP (mg/L)		
			0.11	PM_10_ (mg/L)		
			0.10	Disposal ratio of industrial gas (%)		
			0.12	Pollution burden (person/km^2^)		
	**0.27**	Biodiversity	0.28	High degree of non-living environment	0.48	Temperature of annual average (°C)
					0.52	Quantity of rainfall (mm)
			0.38	Abundance of biological resource	0.31	Protection Wildlife of national and province rank /marsh species
					0.42	Cover ratio of vegetation (%)
					0.27	Biological diversity
			0.34	Degree of social and economic development	0.18	Average fixed assets investment
					0.18	Average net income (dollar)
					0.47	Engle coefficient (%)
					0.17	Urbanization level (%)

**Table 6.** Synthetic eco-environment sensitivity evaluation results for Yicheng City.

**Type of sensitivity**	**Sensitivity index**	**Area (hm^2^)**	**Proportion (%)**	**Feasible land type**
Slight	3	60,497.36	29.49	Feasible zone
Moderate	5	53,317.27	25.99	Feasible zone
High	7	61,789.78	30.12	Dominated zone
Extreme	9	29,540.93	14.4	Protected zone
